# Comparative Analysis of Hybrid Models for Prediction of BP Reactivity to Crossed Legs

**DOI:** 10.1155/2017/2187904

**Published:** 2017-11-26

**Authors:** Gurmanik Kaur, Ajat Shatru Arora, Vijender Kumar Jain

**Affiliations:** ^1^Department of Electrical Engineering, SBBSU, Khiala, District Jalandhar, Punjab 144030, India; ^2^Department of Electrical and Instrumentation Engineering, SLIET, Deemed University (Established by Govt. of India), Longowal, District Sangrur, Punjab 148106, India

## Abstract

Crossing the legs at the knees, during BP measurement, is one of the several physiological stimuli that considerably influence the accuracy of BP measurements. Therefore, it is paramount to develop an appropriate prediction model for interpreting influence of crossed legs on BP. This research work described the use of principal component analysis- (PCA-) fused forward stepwise regression (FSWR), artificial neural network (ANN), adaptive neuro fuzzy inference system (ANFIS), and least squares support vector machine (LS-SVM) models for prediction of BP reactivity to crossed legs among the normotensive and hypertensive participants. The evaluation of the performance of the proposed prediction models using appropriate statistical indices showed that the PCA-based LS-SVM (PCA-LS-SVM) model has the highest prediction accuracy with coefficient of determination (*R*^2^) = 93.16%, root mean square error (RMSE) = 0.27, and mean absolute percentage error (MAPE) = 5.71 for SBP prediction in normotensive subjects. Furthermore, *R*^2^ = 96.46%, RMSE = 0.19, and MAPE = 1.76 for SBP prediction and *R*^2^ = 95.44%, RMSE = 0.21, and MAPE = 2.78 for DBP prediction in hypertensive subjects using the PCA-LSSVM model. This assessment presents the importance and advantages posed by hybrid computing models for the prediction of variables in biomedical research studies.

## 1. Introduction

Accurate measurement of blood pressure (BP) is indispensable for the diagnosis of hypertension at its early stage. Hypertension appears as a top risk factor for life-threatening conditions such as coronary artery disease, stroke, and kidney failure [1]. However, according to a recent editorial in the Hypertension journal of the American Heart Association (AHA), “few measurements in medicine are done as poorly and consistently as BP measurement. Though, there is clear recognition of biological variability, we continue to make decisions largely on measurements taken at random times under poorly controlled conditions” [2]. This observation supports the need to develop novel methods for accurate prediction of BP.

Recommendations of several international organisations including the AHA [3], British Hypertension Society (BHS) [4], and European Society of Hypertension (ESH) [5] revealed that BP is influenced by numerous biological and analytical sources of variation. Biological variations are relative to changes in the individual and are induced by, for instance, emotions, day and night rhythm, seasons, meals, and postures. Analytical variations are derived from the variability of the instrument used, observer bias, and so forth. However, it is not always feasible to control all the factors, but we can minimize their effect by taking them into account in reaching a decision [5].

Correct positioning of a subject's legs is often neglected during BP measurement. As it seems a comfortable position, subjects spontaneously cross their legs at the knees. Several clinical and research studies have been proved that crossing the legs at knee level during BP measurement has a potential effect on the accuracy of measurements. Foster-Fitzpatrick et al. demonstrated a significant increase in BP taken with the legs crossed at the knee level in hypertensive subjects [6]. Peters et al. reported that crossed legs during BP measurement significantly increased systolic BP (SBP) and diastolic BP (DBP) in hypertensive subjects. In healthy volunteers, SBP and DBP increased when legs were crossed at knee level, but the effect was nonsignificant on DBP [7]. Keele-Smith and Price-Daniel, demonstrated that BP was significantly higher when legs were crossed versus uncrossed in a well-senior population [8]. Pinar et al. showed that crossing legs at knee level increased BP readings in hypertensive subjects [9]. Adiyaman et al. found significant increases in BP readings when the legs were crossed at knee level [10]. van Groningen et al. measured BP using a Finometer; they found an increase in BP readings with the legs crossed at knee level [11]. Pinar et al. reported that in hypertensive subjects, BP increased significantly when they crossed their legs [12].

Despite studies confirming the importance of leg position on BP measurement, it is likely that leg position varies markedly in clinical practice and also in published studies [2] and it may result in the misdiagnosis of hypertension or in overestimation of the severity of hypertension and may lead to overly aggressive therapy. Antihypertensive treatment may be unnecessary in the absence of concurrent cardiovascular risk factors [13].

Moreover, there is growing evidence that anthropometric indices are a major determinant of BP. Several studies have been conducted in the past to identify anthropometric characteristics that can be used as markers of BP [14–16]. These studies have explored a significant correlation between BP and anthropometric characteristics of a subject. Therefore, anthropometric characteristics should be considered to attain an accurate measurement of BP reactivity. However, multicollinearity between anthropometric characteristics has also been reported, which may result in “overfitting” of the prediction model [17–19].

The various methods utilized for prediction of biological variables range from the traditional statistical models to the complicated artificial intelligence-based models [20–25]. Recent studies on prediction of BP are as follows: Monte-Moreno presented a system for simultaneous noninvasive estimate of the blood glucose level (BGL), SBP, and DBP using a photoplethysmograph (PPG) and machine learning techniques. Physiological properties including blood viscosity, vessel compliance, hemodynamics, metabolic syndrome, demographic characteristics, and emotional state were used as input variables. The machine learning techniques tested were as follows: ridge linear regression, multilayer perceptron artificial neural network (ANN), support vector machine (SVM), and random forest. The best results were obtained with the random forest technique [26]. Genc proposed a linear stochastic model that integrated a known portion of the cardiovascular system and unknown portion through a parameter estimation to predict evolution of the mean arterial pressure (MAP). The performance of the model was tested on a case study of acute hypotensive episodes (AHEs) on PhysioNet data. They concluded that true positive rates (TPRs) and false positive rates (FPRs) were improved during the prediction period [27]. Forouzanfar et al. presented a novel feature-based ANN for estimation of BP from wrist oscillometric measurements. Unlike previous methods that used the raw oscillometric waveform envelope (OMWE) as input to the ANN, in this paper, they proposed to use features extracted from the envelope. The OMWE was mathematically modeled as a sum of two Gaussian functions. The optimum parameters of the Gaussian functions were found by minimizing the least squares error (LSE) between the model and the OMWE using the Levenberg Marquardt algorithm and were used as input features. The performance of ANN was compared with that of the conventional maximum amplitude algorithm (MAA), adaptive neuro fuzzy inference system (ANFIS), and already-published ANN-based methods. It was found that the proposed approach achieved lower values of mean absolute error (MAE) and standard deviation (*σ*) of error (SDE) in the estimation of BP [28]. Kurylyak et al. estimated the BP from the PPG signal using ANN. Training data were extracted from the multiparameter intelligent monitoring in an intensive care waveform database for better representation of possible pulse and pressure variation. The comparison between estimated and reference values showed better accuracy than the linear regression method [29]. Golino et al. compared the classification tree technique with traditional logistic regression for prediction of BP. Body mass index (BMI), waist circumference (WC), hip circumference (HC), and waist-hip ratio (WHR) were used as predictor variables. Finally, the comparison of the classification tree technique with traditional logistic regression indicated that the former outperformed the latter in terms of predictive power [30].

Hsin-Hsiuang et al. compared logistic regression, SVM, and permanental classification methods in predicting hypertension by using the genotype information. They used logistic regression analysis in the first step to detect significant single-nucleotide polymorphisms (SNPs). In the second step, they used the significant SNPs with logistic regression, SVM, and permanental classification methods for prediction purposes. The results showed that SVM and permanental classification both outperformed logistic regression [31]. Khan et al. proposed SVM for performing the prediction of BP with primary emotions using Facebook status. Current human BP and those belonging to up to six previous primary emotions and BP values with respect to human emotion were given as input variables. The outcome showed that SVM can be prosperously applied for prediction of BP through primary emotions. On the contrary, validations signified that the error statistics of the SVM model marginally outperformed [32]. Barbe et al. developed a logistic regression model to calibrate and correct an oscillometric monitor such that the device better corresponds to the Korotkoff method regardless of the health status of the patient. The model eliminated the systematic errors caused by patients suffering from hyper- or hypotension. They reported that systematic error was reduced by nearly 50% corresponding to the performance specifications of the device [33].

To perform a better training process and improve the forecasting accuracy, hybrid computing models in medical diagnosis are being developed to support physicians in successful decision making regarding clinical admission, early prevention, early clinical diagnosis, and application of clinical therapies by allowing calculation of disease likelihood based on known subject characteristics and clinical test results [34]. The main premise behind developing a hybrid computing model is to exploit the synergy between two or more models, leveraging their benefits and overcoming their respective limitations. The past few years have seen a vast interest in the hybrid computing models that seem to have completely replaced the traditional unisystem approaches. The rationale of using hybrid modeling in biomedical research studies is mainly to obtain fewer important predictor variables, and the selected predictor variables can serve as inputs for the designed prediction model. Hence, hybrid approach can improve the diagnostic accuracy with reduction in complexity of the prediction model [35].

The present study is a continuation of our previous studies [36, 37] dealing with the development of hybrid computing techniques for prediction of BP reactivity to talking and unsupported back. This research work focuses on the development of principal component analysis- (PCA-) based forward stepwise regression (FSWR), ANN, ANFIS, and least squares SVM (LS-SVM) hybrid computing models for prediction of BP reactivity to crossed legs by taking into account the anthropometric markers of BP in normotensive and hypertensive subjects. The prediction accuracy of the developed models was assessed using coefficient of determination (*R*^2^), root mean square error (RMSE), and mean absolute percentage error (MAPE).

## 2. Materials and Methods

### 2.1. Participants

A total of 40 normotensive and 30 hypertensive subjects among the students, staff, and faculty of Sant Longowal Institute of Engineering and Technology, Deemed University, Longowal, Distt. Sangrur, Punjab, INDIA, were included in this study. Participants were aged over 18 years. Exclusion criteria were pregnant subjects, arrhythmic subjects, and the subjects who had a history of any condition that would interfere with positioning of lower extremity of the subjects. The institutional research committee approved the research protocol and all participants gave written informed consent before participation.

### 2.2. Data Collection

A standard questionnaire was administrated for the collection of anthropometric data including age, height, weight, BMI, and mid-upper arm circumference (MUAC) of the participants. The mean and standard deviation (SD) of the collected anthropometric data is given in Table 1.

A specially separated room was used to conduct this study. This ensured minimal interference within the room while the tests were being carried out. The observers involved in the study were trained using the BHS's BP measurement training materials [38].

To eliminate the observer bias, BP was measured using a validated, newly purchased, and fully automated sphygmomanometer OMRON HEM-7203 (OMRON HEALTHCARE Co. Ltd., Kyoto, Japan) that uses the oscillometric method of measurement. The BP monitor is available with a small cuff (17–22 cm), medium cuff (22–32 cm), and large cuff (32–42 cm). BP measurement was preceded by selection of the appropriate size cuff according to the MUAC of the subjects.

Subjects were advised to avoid alcohol, cigarette smoking, coffee/tea intake, and exercise for at least 30 minutes prior to their BP measurement. They were instructed to empty their bladder prior to measurements. Subjects were also instructed to sit upright on a chair with a supported back, kept the feet flat on the floor and the upper arm (under measurement) at heart level, as they are the potential confounding factors. Moreover, they were asked not to talk and move during measurement [3].

After a rest period of 5 minutes [3], the measurements were performed four times repeatedly at an interval of one minute. First measurement was discarded and the average of the last three measurements was taken into account. Subsequently, the legs were crossed at the knees and after four minutes, the same measurement protocol was repeated. All measurements were obtained under similar measurement conditions except for the different leg positions. And the measurement protocol was repeated for 7 days.

### 2.3. Experimental Methods

#### 2.3.1. PCA

PCA is the first step of counteracting multicollinearity. It is a dimension reduction technique that does not take the correlation between the input variables into account. Thus, PCA is considered as an unsupervised dimension reduction method [39–41]. To evaluate the influence of each input variable in the PCA, varimax rotation was used to obtain values of rotated factor loadings. The Kaiser-Meyer-Olkin (KMO) measure of sampling adequacy and Barlett's test of sphericity were used to check the suitability of data for application of PCA [42–45].

#### 2.3.2. FSWR

FSWR is a traditional statistical modeling technique used for developing an optimum prediction model by extracting the best anthropometric characteristics or predictor variables depending upon their statistical significance or probability (*p*) value. It starts with an empty prediction model and adds one anthropometric predictor variables at a time. The first predictor variable included in the model has the highest correlation with the independent variable *y*. The second variable included is the one which has the highest correlation with *y*, after *y* has been adjusted for the effect of the first predictor variable. This process terminates when the last variable entering the model has insignificant regression coefficient [46].

#### 2.3.3. ANN

To achieve the best architecture of ANN, various structures of feed-forward ANN with different numbers of hidden layers and neurons in each hidden layer were investigated. Finally, in light of the performance indices obtained from investigations, an ANN structure with two hidden layers and six nodes in each hidden layer was selected for further analysis. In addition, the architecture of ANN also consisted of one input layer with four input nodes (representing four PCs) and one output layer with one output node (representing BP reactivity to crossed legs). The choice of hyperbolic tangent sigmoid activation function for hidden layer and linear activation function for output layer trained the network in lesser number of epochs with better performance criteria and also yielded the best outcome predictions. The back propagation learning algorithm based on the Levenberg-Marquardt technique was used to find the local minimum of the error function. It blends the steepest descent method and the Gauss-Newton algorithm and inherits the speed advantage of the Gauss-Newton algorithm and the stability of the steepest descent method. It is more powerful and faster than the conventional gradient descent technique [47, 48].

#### 2.3.4. ANFIS

A Sugeno-type FIS model was developed using “genfis1” with grid partitioning on data for prediction of BP reactivity to crossed legs. Different ANFIS parameters including numbers of membership functions (MFs) and types of input and output MF were tested to achieve the perfect training and maximum prediction accuracy. Input membership function “psigmf” and output membership function “linear” were used to develop the prediction model [49].

Other parameters of the trained ANFIS model were as follows: number of MFs = 16, number of nodes = 55, number of linear parameters = 80, number of nonlinear parameters = 32, total number of parameters = 112, and number of fuzzy rules = 16.

#### 2.3.5. LS-SVM

The most important steps to develop a LS-SVM model are as follows: selection of a kernel and its parameters. After many experimental observations, radial basis function (RBF) kernel and grid search optimization algorithm (with 2-fold cross-validation) were selected to obtain the optimal combination of regularization parameter (*γ*) and squared bandwidth (*σ*^2^) [50, 51].

## 3. Results

### 3.1. Effect of Crossed Legs on BP

The results of the paired *t*-test demonstrated a statistically significant higher SBP with crossed legs (mean difference ± SD = 5.838 ± 2.5919, *p* < 0.001) in normotensive subjects, but there was no significant difference between DBP measurements (mean difference ± SD = 0.0037 ± 0.0126, *p* = 0.0737). In hypertensive subjects, both SBP (mean difference ± SD = 10.3524 ± 4.5844, *p* < 0.001) and DBP (mean difference ± SD = 6.1704 ± 1.8531, *p* < 0.001) were significantly different when legs were crossed at knee level. These results are consistent with the recommendations of the AHA council for BP measurement in humans and experimental animals [3].

### 3.2. Multicollinearity Diagnostic

A visual inspection of the Pearson's correlation coefficients revealed the existence of multicollinearity, as correlation coefficient > 0.6 [52], between pairs of anthropometric characteristics, in normotensive and hypertensive individuals, as shown in Table 2.

### 3.3. Application of PCA on BP Data

In the next step, PCA was used to omit the multicollinearity between pairs of anthropometric characteristics and simplify the complexity of the relationship between them [53].

To verify the applicability of PCA, Bartlett's test of sphericity was applied [54]. A high value of chi square (*χ*^2^), for normotensive (*χ*^2^ = 231.012, DF = 10, *p* < 0.0001) and hypertensive (*χ*^2^ = 119.48, DF = 10, *p* < 0.0001) individuals implied that PCA is applicable to our data set. The value of KMO was also greater than 0.6 for normotensive (0.63) and hypertensive (0.75) individuals, which indicates that our sample size is enough to apply PCA [55].

Out of 5 PCs, only the first four PCs (PC1–PC4), explaining more than 5% of variations, were retained for further analysis. In normotensive subjects, the selected PCs explained 99.8% of the total variation. Variance proportions explained by PC1, PC2, PC3, and PC4 were found as 71.84%, 16.58%, 6.34%, and 5.04%, respectively. In hypertensive subjects, the selected PCs explained 98.04% of the total variation. Variance proportion accounted for by PC1, PC2, PC3, and PC4 was estimated to be 61.10%, 22.5%, 8.78%, and 5.66%, respectively. Loadings of anthropometric characteristics after varimax rotation give an indication of the extent to which the original variables are influential in forming new variables. For both normotensive and hypertensive subjects, weight and BMI were the characteristics having the highest correlation with PC1 and height had the highest correlation with PC2.

Moreover, Pearson's correlation between pairs of PCs, as shown in Table 3, indicates that the problem of multicollinearity presented in Table 2 is solved as there is no significant relationship between any pair of PCs in the correlation table (correlation coefficient < 0.6).

To develop PCA-based prediction models, principal score values obtained from the principle score coefficients were used as independent variables and BP reactivity was used as dependent variable. Moreover, 80% data were used for training while the entire data set was used for testing. Data were normalized before training to achieve more accurate predictions. MATLAB (version 7.5) was used to develop the prediction models.

### 3.4. PCA-Based FSWR (PCA-FSWR)

**Figure 1:**
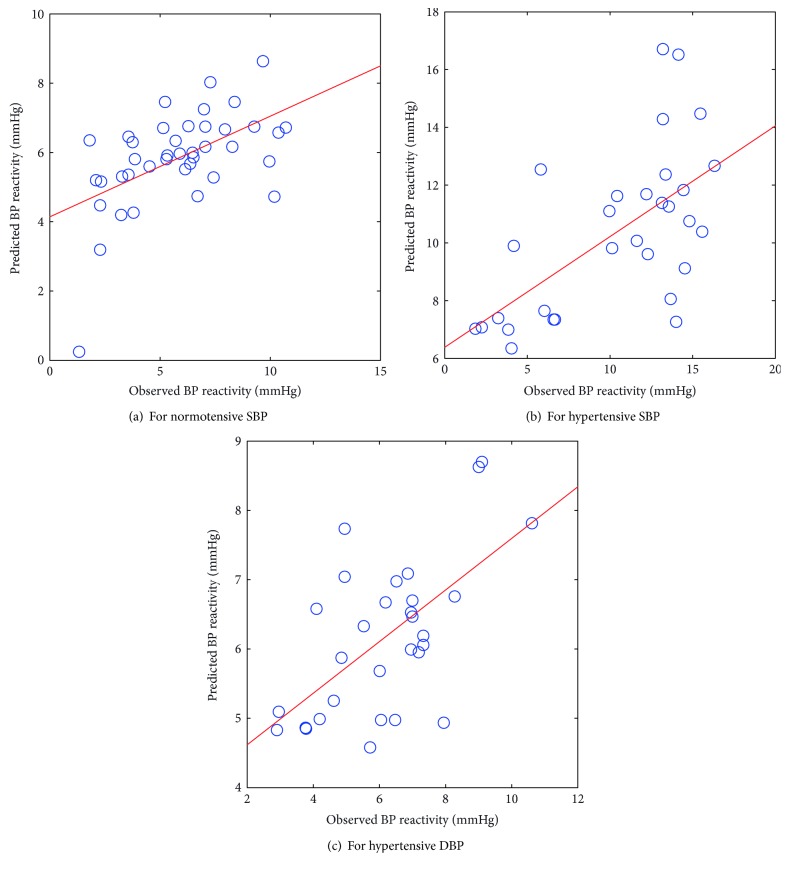
Scatter plot between observed and predicted values of BP reactivity using the PCA-FSWR model.

When probabilities were taken into consideration, the regressions of standardized SBP reactivity on PC1 (composed of weight and BMI) were found statistically significant in normotensive subjects. Whereas, PC3 (composed of age) was found statistically significant for SBP and DBP reactivity in hypertensive subjects. Figures 1(a)–1(c) show the scatter plot between the observed and predicted values of BP reactivity from the PCA-FSWR model in normotensive and hypertensive subjects.

The final model equations for prediction of BP reactivity in normotensive and hypertensive subjects are given as follows:

(a) Model equation obtained for prediction of SBP reactivity in normotensive subjects:
  (1) $$\begin{array}{c} SBP\;reactivity=5.8381-1.8514\left(PC1\right). \end{array}$$
(b) Model equation obtained for prediction of SBP reactivity in hypertensive subjects:
  (2) $$\begin{array}{c} SBP\;reactivity=10.3524-1.6246\left(PC3\right). \end{array}$$
(c) Model equation obtained for prediction of DBP reactivity in hypertensive subjects:
  (3) $$\begin{array}{c} DBP\;reactivity=6.1704-0.6467\left(PC3\right). \end{array}$$

### 3.5. PCA-Based ANN (PCA-ANN)

**Figure 2:**
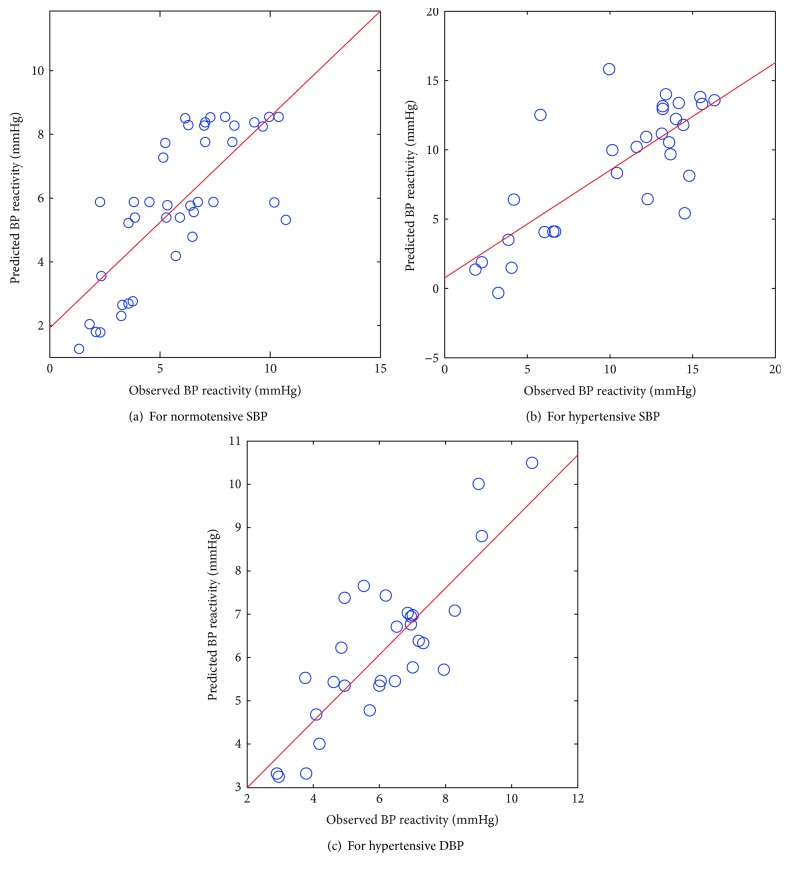
Scatter plot between observed and predicted values of BP reactivity using the PCA-ANN model.

The scatter plots between the observed and predicted values of BP reactivity from the PCA-ANN model, as illustrated in Figures 2(a)–2(c), although revealed marked deviations, but they were smaller than those from the PCA-FSWR model.

### 3.6. PCA-Based ANFIS (PCA-ANFIS)

**Figure 3:**
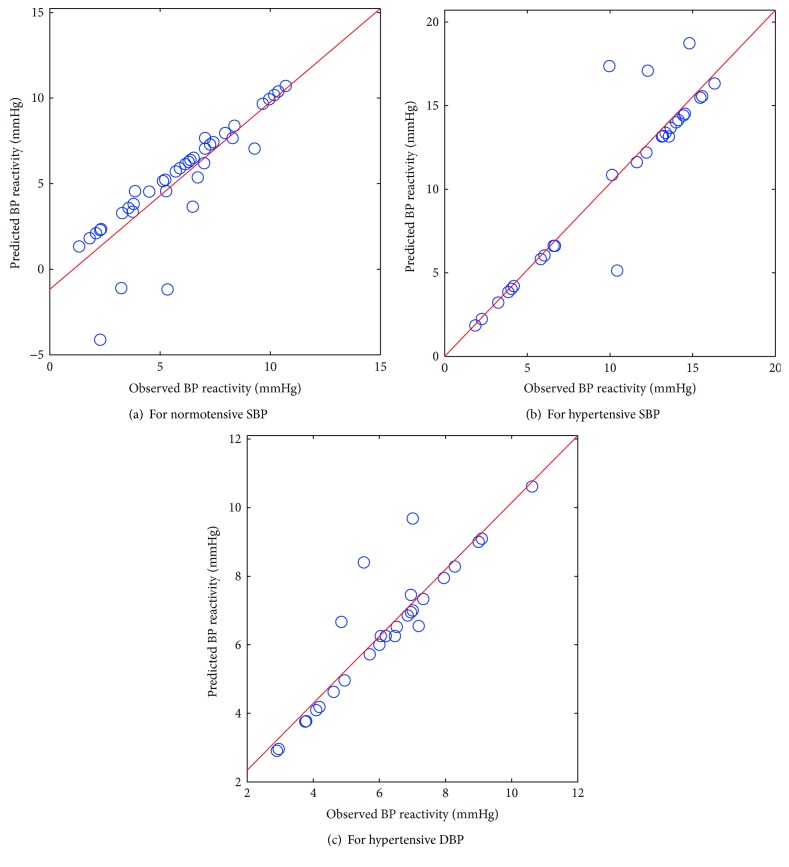
Scatter plot between observed and predicted values of BP reactivity using the PCA-ANFIS model.

As presented in Figures 3(a)–3(c), the scatter plots plotted between observed and predicted values of BP reactivity from the PCA-ANFIS model clearly demonstrate improvements in predicted values as compared to those of the performance of the PCA-FSWR and PCA-ANN prediction models.

### 3.7. PCA-Based LS-SVM (PCA-LS-SVM)

The optimal values of regularization parameter (*γ*) and squared bandwidth (*σ*^2^) obtained from the developed PCA-LS-SVM model are as follows:

1. *γ* = 200, *σ*^2^ = 0.53 (for prediction of SBP reactivity in normotensive subjects)
2. *γ* = 253.0920, *σ*^2^ = 0.0782 (for prediction of SBP reactivity in hypertensive subjects)
3. *γ* = 1.0635*e* + 004, *σ*^2^ = 0.0148 (for prediction of DBP reactivity in hypertensive subjects)

**Figure 4:**
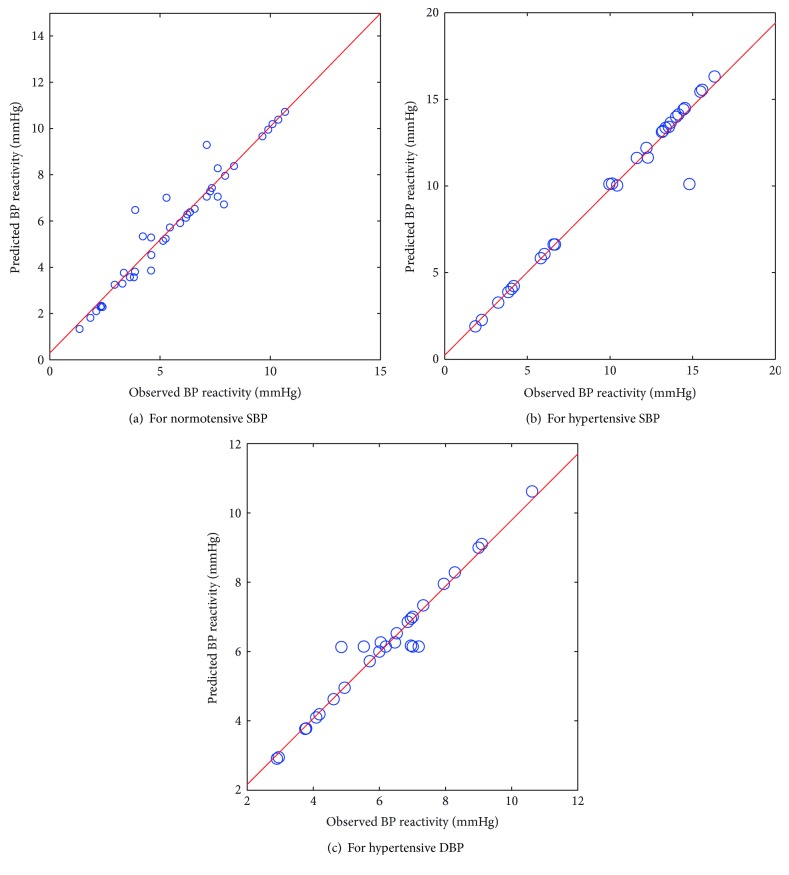
Scatter plot between observed and predicted values of BP reactivity using the PCA-LS-SVM model.

The scatter plots between the observed and predicted values of BP reactivity from PCA-LS-SVM as shown in Figures 4(a)–4(c) revealed the best predicted values when compared to predictions of the PCA-FSWR, PCA-ANN, and PCA-ANFIS models.

The comparison of statistical indices of the models, as shown in Table 4, reveals that the PCA-LS-SVM model has the highest value of *R*^2^ and lowest value of RMSE and MAPE for prediction of BP reactivity to crossed legs in normotensive and hypertensive subjects.

## 4. Discussion

Accurate prediction of BP is integral to successful decision making and leads to better patient care. Overestimation of BP would increase the number of patients with hypertension. They may experience adverse effects of medication and have increased insurance and treatment cost. Furthermore, the inaccurate labeling leads to an increased perception of disease and absenteeism from work [56].

The marked elevation in BP with the crossed leg position may be due to isometric activity of the leg muscles. Isometric activity increases vascular resistance or total peripheral resistance (TPR) and BP [57]. Another explanation for the significant rise in BP with the crossed legs is translocation of blood volume from the dependent vascular beds in the legs to the central thoracic compartment that causes a high stroke volume, as cardiac output is determined by the stroke volume multiplied by heart rate. Therefore, an increase in stroke volume causes an increase in cardiac output [6].

Evidently, this work demonstrates that crossed legs in sitting position significantly elevated SBP of normotensive subjects and SBP and DBP of hypertensive subjects. Similar conclusions were found by previous studies [6–12].

Furthermore, PCA-based hybrid computing models for predictions of BP reactivity to crossed legs are proposed in this paper. To the best of our knowledge, this is the first study that focused specifically on prediction of BP reactivity to crossed legs using the PCA-FSWR, PCA-ANN, PCA-ANFIS, and PCA-LS-SVM models. Therefore, the results were compared with indirectly related prediction studies, as shown in Table 5.

In all studies, the higher performance of the soft computing models was sourced from a greater degree of robustness and fault tolerance than traditional models. The results of present research work illustrated that the PCA-LS-SVM hybrid model obtained the best prediction results because LS-SVM is firmly based on the theory of statistical learning; therefore, it can attain a global optimal solution and has good generalization ability and low dependency on sample data.

The present study has a number of merits. We used small, medium, and large size cuffs to cover the entire MUAC range demanded by participants. Inappropriate cuff size results in underestimation or overestimation of BP. Moreover, to strengthen the accuracy of measurements, we took the mean of three readings per leg position for seven days [3].

However, any single comparison between the prediction models might not reliably represent the true end results. It is essential to assess the performance of prediction models in external validation studies using larger database.

## 5. Conclusions

This paper has detailed an examination of hybrid computing models in an effort to predict BP reactivity to crossed legs using anthropometric predictor variables. By eliminating the multicollinearity problem, PCA provided more objective interpretation of anthropometric predictor variables used for prediction. Then, the PCA-FSWR, PCA-ANN, PCA-ANFIS, and PCA-LS-SVM models were tested for prediction of BP from PCs. It was found that the PCA-LS-SVM model achieves substantial improvements in terms of *R*^2^, RMSE, and MAPE compared with all the other models. This research work may provide valuable reference for researchers and engineers who apply hybrid computing approaches for modeling biological variables. The results may also be helpful to physicians in making more accurate diagnosis of hypertension in clinical practice. Our future research is targeted to study an ensemble approach by combining the outputs of different hybrid techniques with more predictor variables. In addition, future research work will address using an ensemble approach by combining the outputs of different hybrid models with more predictor variables.

## Figures and Tables

**Table 1:** Descriptive statistics of anthropometric characteristics of study samples.

Anthropometric characteristics	Normotensives		Hypertensives	
	Mean	SD	Mean	SD
Age (years)	23.1	1.24	42.83	6.665
Height (cm)	1.61	0.03	1.583	0.035
Weight (kg)	55.96	7.29	62.48	10.89
BMI (kg/m^2^)	21.55	2.504	23.57	3.497
MUAC (cm)	26.56	2.45	26.72	2.4

**Table 2:** Pearson's correlation coefficients between each pair of anthropometric characteristics in normotensive and hypertensive subjects.

Anthropometric characteristics	Height	Weight	BMI	MUAC
Age (years)	0.535 **0.113**	0.784^∗^ **0.598**	0.701^∗^ **0.509**	0.668^∗^ **0.585**
Height (cm)		0.543 **0.165**	0.237 **0.305**	0.619^∗^ **0.021**
Weight (kg)			0.934^∗^ **0.885**^∗^	0.743^∗^ **0.767**^∗^
BMI (kg/m^2^)				0.617^∗^ **0.691**^∗^

∗ indicates *p* < 0.001; bold values indicate correlations between anthropometric characteristics of hypertensive subjects.

**Table 3:** Pearson's correlation coefficient between each pair of PCs in normotensive and hypertensive subjects.

PC	PC2	PC3	PC4
*PC1*	−0.00000225 **0.00000878**	0.0000000798 **0.00000423**	−0.0000167 **0.00000659**
*PC2*		−7.237*e*−016 **0.00000919**	5.808*e*−016 **0.0000142**
*PC3*			−7.557*e*−017 **0.0000175**

Bold values indicate correlation in anthropometric characteristics of hypertensive subjects.

**Table 4:** Statistical indices for the proposed models.

Model	Normotensive subjects			Hypertensive subjects					
	SBP			SBP			DBP		
	*R* ^2^ (%)	RMSE	MAPE (%)	*R* ^2^ (%)	RMSE	MAPE (%)	*R* ^2^ (%)	RMSE	MAPE (%)
PCA-FSWR	29.05	2.21	40.33	38.35	3.66	48.35	37.21	1.49	22.72
PCA-ANN	55.67	0.67	26.25	60.11	0.74	30.39	67.91	0.57	14.63
PCA-ANFIS	75.42	0.67	17.39	84.81	0.44	6.74	84.26	0.44	5.06
PCA-LS-SVM	93.16	0.27	5.71	96.46	0.19	1.76	95.44	0.21	2.78

**Table 5:** Comparison of results with other studies.

Ref.	Model developed	Predicted parameter	Results
[26]	Ridge linear regression, ANN, SVM, and random forest	BGL, BP	Random forest technique outperformed ridge linear regression, ANN, and SVM. *R*^2^ = 0.91% (SBP), *R*^2^ = 0.89% (DBP), and *R*^2^ = 0.90% (BGL)
[28]	ANN (raw input), ANN (feature based), MAA, and ANFIS (feature based)	SBP, DBP	ANN (feature based) achieved the best performance compared to other models. For SBP predictions: MAE = 6.28, SDE = 8.58. For DBP predictions: MAE = 5.73, SDE = 7.33
[29]	ANN	SBP, DBP	The experimental results confirmed the correctness of the ANN when compared with the linear regression model. Mean ± *σ*: SBP: 3.80 ± 3.46, DBP: 2.21 ± 2.09. Relative error: SBP: 3.48 ± 3.19. DBP: 3.90 ± 3.51
[32]	SVM with RBF and polynomial kernel	SBP, DBP	SVM (RBF kernel) outperformed SVM (polynomial kernel). Coefficient of correlation (*R*) = 0.97 (SBP), 0.96 (DBP). RMSE = 6.94 (SBP), and 5.78 (DBP). Scatter index (SI) = 22.34 (SBP), 22.79 (DBP)
[36]	PCA-ANN, PCA-ANFIS, and PCA-LS-SVM	SBP, DBP	PCA-LS-SVM outperformed PCA-ANN and PCA-ANFIS. For normotensive subjects: SBP: *R*^2^ = 95.42%, RMSE = 0.21, and MAPE = 5.88%. DBP: *R*^2^ = 94.22%, RMSE = 0.24, and MAPE = 4.05%. For hypertensive subjects: SBP: *R*^2^ = 98.76%, RMSE = 0.11, and MAPE = 0.88%. DBP: *R*^2^ = 98.78%, RMSE = 0.11, and MAPE = 0.84%
[37]	PCA-SWR, PCA-ANN, PCA-ANFIS, and PCA-LS-SVM	DBP	PCA-LS-SVM outperformed PCA-FSWR, PCA-ANN, and PCA-ANFIS. For normotensive subjects: *R*^2^ = 98.49%, RMSE = 0.1243, and MAPE = 3.01%. For hypertensive subjects: *R*^2^ = 95.95%, RMSE = 0.2013, and MAPE = 2.9%
[58]	ANN, ANFIS, and SVM	River flow in the semiarid mountain region	In comparing the results of the ANN, ANFIS, and SVM models, it was seen that the values of *R*, RMSE, mean absolute relative error (MARE), and Nash-Sutcliffe (NS) of the SVM model were higher than those of ANN and ANFIS for all combinations of input data
[59]	ANN, ANFIS	To predict depths-to-water table one month in advance, at three wells located at different distances from the river	Both models can be used with a high level of precision to the model water tables without a significant effect of the distance of the well from the river, as model precision expressed via RMSE was roughly the same in all three cases (0.14154–0.15248). *R* varied from 0.91973 to 0.9623 and coefficient of efficiency (COE) from 0.84588 to 0.92586
[60]	ANN, ANFIS, and SVM	Longitudinal dispersion coefficient (LDC)	The SVM model was found to be superior (*R*^2^ = 90%) in predicting LDC due to low uncertainty as compared with those in the ANN (*R*^2^ = 82%) and ANFIS (*R*^2^ = 83%) models, while the ANFIS model performed better than the ANN model
[61]	Multilayer perceptron (MLP), ANN, fuzzy genetic (FG), LS-SVM, multivariate adaptive regression spline (MARS), ANFIS, multiple linear regression (MLR), and Stephens and Stewart models (SS)	Evaporation in different climates	The accuracies of the applied models were rank as: MLP, GRNN, LSSVM, FG, ANFIS-GP, MARS, and MLR
Present study	PCA-FSWR, PCA-ANN, PCA-ANFIS, and PCA-LS-SVM	BP reactivity to crossed legs	PCA-LS-SVM outperformed PCA-FSWR, PCA-ANN, and PCA-ANFIS. For normotensive subjects: SBP: *R*^2^ = 93.16%, RMSE = 0.27, and MAPE = 5.71%. For hypertensive subjects: SBP: *R*^2^ = 96.46%, RMSE = 0.19, and MAPE = 1.76%. DBP: *R*^2^ = 95.44%, RMSE = 0.21, and MAPE = 2.78%

## References

[1] George S. S., Loanni A. B. (2011). Home blood pressure monitoring in the diagnosis and treatment of hypertension: a systematic review. *American Journal of Hypertension*.

[2] Jones D. W., Hall J. E. (2008). Hypertension: pathways to success. *Hypertension*.

[3] Pickering T. G., Hall J. E., Appel L. J. (2005). Recommendations for blood pressure measurement in humans and experimental animals: part 1: blood pressure measurement in humans: a statement for professionals from the Subcommittee of Professional and Public Education of the American Heart Association Council on high blood pressure research. *Circulation*.

[4] Williams B., Poulter N. R., Brown M. J. (2004). Guidelines for management of hypertension: report of the fourth working party of the British hypertension society 2004-BHS IV. *Journal of Human Hypertension*.

[5] O’Brien E., Asmar R., Beilin L. (2003). European Society of Hypertension recommendations for conventional, ambulatory and home blood pressure measurement. *Journal of Hypertension*.

[6] Foster-Fitzpatrick L., Ortiz A., Sibilano H., Marcantonio R., Braun L. T. (1999). The effects of crossed leg on blood pressure measurement. *Nursing Research*.

[7] Peters G. L., Binder S. K., Campbell N. R. (1999). The effect of crossing legs on blood pressure: a randomized single blind cross-over study. *Blood Pressure Monitoring*.

[8] Keele-Smith R., Price-Daniel C. (2001). Effects of crossing legs on blood pressure measurement. *Clinical Nursing Research*.

[9] Pinar R., Sabuncu N., Oksay A. (2004). Effects of crossed leg on blood pressure. *Blood Pressure*.

[10] Adiyaman A., Tosun N., Elving L. D., Deinum J., Lenders J. W. M., Thien T. (2007). The effect of crossing legs on blood pressure. *Blood Pressure Monitoring*.

[11] van Groningen L. F., Adiyaman A., Elving L., Thien T., Lenders J. W., Deinum J. (2008). Which physiological mechanism is responsible for the increase in blood pressure during leg crossing?. *Journal of Hypertension*.

[12] Pinar R., Watson R. (2010). The effect of crossing legs on blood pressure in hypertensive subjects. *Journal of Clinical Nursing*.

[13] Vidit D. G., Lang R. S., Seballos R. J., Misra-Hebert A., Campbell J., Bena J. F. (2010). Taking blood pressure: too important to trust to humans?. *Cleveland Clinic Journal of Medicine*.

[14] Fuchs F. D., Gus M., Moreira L. B. (2005). Anthropometric indices and the incidence of hypertension: a comparative analysis. *Obesity Research*.

[15] Moser D. C., Giuliano I. C., Titski A. C., Gaya A. R., Coelho-e-Silva M. J., Leite N. (2013). Anthropometric measures and blood pressure in school children. *Jornal de Pediatria*.

[16] Mariko I., Masahide M., Eiji Y. (2014). Body mass index, blood pressure, and glucose and lipid metabolism among permanent and fixed-term workers in the manufacturing industry: a cross-sectional study. *BMC Public Health*.

[17] Doll S., Paccaud F., Bovet P., Burnier M., Wietlisbach V. (2002). Body mass index, abdominal adiposity and blood pressure: consistency of their association across developing and developed countries. *International Journal of Obesity and Related Metabolic Disorders*.

[18] Bose K., Ghosh A., Roy S., Gangopadhyay S. (2003). Blood pressure and waist circumference: an empirical study of the effects of waist circumference on blood pressure among Bengalee male jute mill workers of Belur, West Bengal, India. *Journal of Physiological Anthropology and Applied Human Science*.

[19] Baba R., Koketsu M., Nagashima M., Inasaka H., Yoshinaga M., Yokota M. (2007). Adolescent obesity adversely affects blood pressure and resting heart rate. *Circulation Journal*.

[20] Gurmanik K., Ajat Shatru A., Vijender J. K. (2009). Multi-class support vector machine classifier in EMG diagnosis. *WSEAS Transactions on Signal Processing*.

[21] Mohebian M. R., Marateb H. R., Mansourian M., Mananas M. A., Mokarian F. (2017). A hybrid computer-aided-diagnosis system for prediction of breast cancer recurrence (HPBCR) using optimized ensemble learning. *Computational and Structural Biotechnology Journal*.

[22] Thomas J., Theresa P. R. Human heart disease prediction system using data mining techniques.

[23] Steyerberg E. W., Vickers A. J., Cook N. R. (2010). Assessing the performance of prediction models: a framework for some traditional and novel measures. *Epidemiology*.

[24] Vezzoli M., Ravaggi A., Zanotti L. (2017). RERT: a novel regression tree approach to predict extrauterine disease in endometrial carcinoma patients. *Scientific Reports*.

[25] Johnson P., Vandewater L., Wilson W. (2014). Genetic algorithm with logistic regression for prediction of progression to Alzheimer’s disease. *BMC Bioinformatics*.

[26] Monte-Moreno E. (2011). Non-invasive estimate of blood glucose and blood pressure from a photoplethysmograph by means of machine learning techniques. *Artificial Intelligence in Medicine*.

[27] Genc S. Prediction of mean arterial blood pressure with linear stochastic models.

[28] Forouzanfar M., Dajani H. R., Groza V. Z., Bolic M., Rajan S. (2011). Feature-based neural network approach for oscillometric blood pressure estimation. *IEEE Transaction on Instrumentation and Measurement*.

[29] Kurylyak Y., Lamonaca F., Grimaldi D. A neural network-based method for continuous blood pressure estimation from a PPG signal.

[30] Golino H. F., Amaral S. B., Duarte S. F. P. (2014). Predicting increased blood pressure using machine learning. *Journal of Obesity*.

[31] Huang H. H., Xu T., Yang J. (2014). Comparing logistic regression, support vector machines, and permanental classification methods in predicting hypertension. *BMC Proceedings*.

[32] Khan S. M. U., Manzoor J. S., Lee S. U. J. Predicting student blood pressure by support vector machine using Facebook.

[33] Barbe K., Kurylyak Y., Lamonaca F. (2014). Logistic ordinal regression for the calibration of oscillometric blood pressure monitors. *Biomedical Signal Processing and Control*.

[34] Floares A. G. (2010). Using computational intelligence to develop intelligent clinical decision support systems. *Computational Intelligence Methods for Bioinformatics and Biostatistics*.

[35] Kala R., Shukla A., Tiwari R. Comparative analysis of intelligent hybrid systems for detection of PIMA Indian diabetes.

[36] Gurmanik K., Ajat Shatru A., Vijender J. K. (2014). Prediction of BP reactivity to talking using hybrid soft computing approaches. *Computational and Mathematical Methods in Medicine*.

[37] Gurmanik K., Ajat Shatru A., Vijender J. K. (2015). Using hybrid models to predict blood pressure reactivity to unsupported back based on anthropometric characteristics. *Frontiers of Information Technology & Electronic Engineering*.

[38] British Hypertension Society (1998). *Blood Pressure Measurement (CD-ROM)*.

[39] Beatriz H., Rafeal P., Marisol V., Enrique B., Manuel F. J., Luis F. (2000). Temporal evolution of groundwater composition in an alluvial aquifer (Pisuerga river, Spain) by principal component analysis. *Water Research*.

[40] Pett M. A., Lackey N. R., Sullivan J. J. (2003). *Making Sense of Factor Analysis: The Use of Factor Analysis for Instrument Development in Health Care Research*.

[41] Handan C., Nilsun D., Arzu K., Syddyk K. (2005). Use of principal component scores in multiple linear regression models for prediction of *Chlorophyll-a* in reservoirs. *Ecological Modelling*.

[42] Jackson D. A. (1993). Stopping rules in principal components analysis: a comparison of heuristical and statistical approaches. *Ecology*.

[43] Tabachnick B. G., Fidell L. S. (2006). *Using Multivariate Statistics*.

[44] Noori R., Abdoli M. A., Ghasrodashti A. A., Ghazizade M. J. (2009). Prediction of municipal solid waste generation with combination of support vector machine and principal component analysis: a case study of Mashhad. *Environmental Progress & Sustainable Energy*.

[45] Wackernagel H. (2010). *Multivariate Geostatistics: An Introduction with Applications*.

[46] Chatterjee S., Hadi A. S., Price B. (2004). *Regression Analysis by Example*.

[47] Hagan M. T., Menhaj M. B. (1994). Training feedforward networks with the Marquardt algorithm. *IEEE Transactions on Neural Networks*.

[48] Basheer I. A., M. Hajmeer M. (2000). Artificial neural networks: fundamentals, computing, design, and application. *Journal of Microbiological Methods*.

[49] Jang J. S. R. (1993). ANFIS: adaptive-network-based fuzzy inference system. *IEEE Transactions on Systems, Man and Cybernetics*.

[50] Smola A. J., Scholkopf B. (1998). On a kernel based method for pattern recognition, regression, approximation and operator inversion. *Algorithmica*.

[51] Suykens J. A. K., Van Gestel T., De Brabanter J., De Moor B., Vandewalle J. (2002). *Least Squares Support Vector Machines*.

[52] Benesty J., Chen J., Huang Y. (2008). On the importance of the Pearson correlation coefficient in noise reduction. *IEEE Transactions on Audio, Speech and Language Processing*.

[53] Jolliffe I. T. (2002). *Principal Component Analysis*.

[54] Pares-Neto P. R., Jackson D. A., Somers K. M. (2005). How many principal components? Stopping rules for determining the number of non-trivial axes revisited. *Computational Statistics & Data Analysis*.

[55] Kaiser H. F. (1960). The application of electronic computers to factor analysis. *Educational and Psychological Measurement*.

[56] Handler J. (2009). The importance of accurate blood pressure measurement. *The Permanente Journal*.

[57] Hisdal J., Toska K., Flatebo T., Waaler B., Walloe L. (2004). Regulation of arterial blood pressure in humans during isometric muscle contraction and lower body negative pressure. *European Journal of Applied Physiology*.

[58] Zhibin H., Xiaohu W., Hu L., Jun D. (2014). A comparative study of artificial neural network, adaptive neuro fuzzy inference system and support vector machine for forecasting river flow in the semiarid mountain region. *Journal of Hydrology*.

[59] Nevenka D., Milka D., Ruzica S. (2015). Comparison of groundwater level models based on artificial neural networks and ANFIS. *The Scientific World Journal*.

[60] Roohollah N., Zhiqiang D., Amin K., Fatemeh T. K. (2016). How reliable are ANN, ANFIS, and SVM techniques for predicting longitudinal dispersion coefficient in natural rivers?. *Journal of Hydraulic Engineering*.

[61] Lunche W., Ozgur K., Mohammad Z. K., Yiqun G. (2016). Comparison of six different soft computing methods in modeling 2 evaporation in different climates. *Hydrology and Earth System Sciences Discussions*.

